# The Biology of Cancer: A New Approach

**Published:** 1977-03

**Authors:** P. R. J. Burch


*Br. J. Cancer* (1977) **35**, 388.

### *Letters to the Editor*

### THE BIOLOGY OF CANCER: A NEW APPROACH

SIR,—Much could be written about the principles of reviewing. But is it not stretching some principle to its limit when a review is largely confined to one part, of one chapter, of a 12-chapter book? This is what J. Peto does in reviewing my book, " The Biology of Cancer: A New Approach."

He refers quite reasonably to a " hundredfold increase in reported rates " of lung cancer during this century. The Royal College of Physicians argued in their 1971 report *Smoking and Health Now*: "The chief reason for rejecting the genetic hypothesis (of the association between smoking and lung cancer) is its inability to account for the enormous rise in death rates from lung cancer in the past half century." They dismissed the idea " That the increase is fictitious, and due to doctors having mistaken lung cancer for other diseases in the earlier part of this century " because the . . . " rise has been far greater in men than in women. . .".

Since that report, I have demonstrated, both in the *Lancet* and in my book (Fig. 10.14), that the recorded increases in death-rates, generally larger in men than in women, were nevertheless remarkably synchronous in the two sexes, although women took up the habit of cigarette smoking some 30 years later than men (Todd, 1972). Furthermore, the recorded rises have been so large in both sexes that only a small proportion, at the most, could be attributed to the effects of tobacco.

From an extended correspondence with members of the Royal College committee preparing a revised report, and in particular with Professor C. M. Fletcher, I am gratified to learn that they have now abandoned their 1971 position. They concede that under-diagnosis of lung cancer was severe at the beginning of the century. However, in an attempt to salvage some support from the wreck of the secular trends, R. Peto (1976) has recently shifted the focus of interest from absolute rates to the change with time in the sex ratio of death rates for lung cancer. These trends, he believes, really do demonstrate the causal effects of smoking.

In the course of his review, J. Peto takes up this theme from R. Peto and cites sex ratios which he also seems to believe establish the carcinogenic action of tobacco smoke beyond dispute. He comments: " I eagerly await Professor Burch's unconvincing *ad hoc* argument ". It would be churlish of me to deprive him and your readers of a response, although attention to pages 324 to 327 of my book should have rendered it unnecessary.

It is customary to compare quantitative observations with quantitative predictions from hypothesis. Adopting this routine procedure in my book, I calculated the expected increase in the levels of lung cancer over the period 1901–05 to around 1956 assuming: (i) the causal hypothesis of the association between smoking and lung cancer; (ii) the smoking habits of men and women in the United Kingdom as given by Todd (1972); (iii) the presumptive " dose-response " relations determined by Doll and Hill (1964) from their study of British male doctors; and (iv) Hammond's (1966) presumptive " dose-response " relations for U.S. women—in the absence of suitable data for British women. (Curiously, Peto asserts that I do not deal " with the central predictions " of either my own or the conventional models.)

For men, the expected increase proved to be a factor of about 2·9 only, and for women, about 2·3. (The reason for the surprisingly small expected increase in men is that the consumption of non-cigarette tobacco at the end of the nineteenth century—mainly pipe tobacco—was some 3·7 times higher than in 1956.) J. Peto states: " The enormous increase in recorded lung cancer deaths this century, although *exaggerated* by improved diagnosis, is *largely due to cigarette smoking* ". (My italics.) Peto has neglected simple arithmetic. By no plausible stretch of the " conventional model " can cigarette smoking account for as much as 10% of the recorded increase in age-standardized rates in males; the best estimate from published data lies between 2 and 3%.

From the 2 factors of expected increase, 2·9 and 2·3, and the sex ratio (M/F) of standardized death rates from lung cancer in 1901–05 (about 1·24), the predicted sex ratio in 1956 becomes: $1{\cdot}24 \times 2{\cdot}9/2{\cdot}3$ or about 1·56. Peto quotes observed ratios (for an undefined age-range, but probably 50–54) of 8·9 for 1953 and 5·6 for 1963.

The " agreement " between expected and observed ratios leaves something to be desired. I have, however, known larger discrepancies than this to be " resolved " by ingenious *post hoc* devices. Perhaps the " robustness " of the " conventional model " will prove equal to the task?

As things stand, the calculations given already in Chapter 10 of my book appear to dispose of the claims of J. and R. Peto, without resort to any *ad hoc* arguments. I must hasten to add, as I did in my book, that the analysis of recorded secular trends in death rates—together with *post mortem* studies—shows that the increases were largely the result of diagnostic error and, perhaps, non-cigarette-associated genuine increases. However, because these confounding effects are so large, the analysis of secular trends does not dispose of the hypothesis that smoking causes lung cancer. A genuine cigarette-caused increase might well be concealed in the overwhelmingly larger recorded rise.

According to J. Peto my " . . . style is well illustrated by the discussion of inhalation ". He states (correctly): " Among heavy smokers, inhalers suffer lung cancer rates similar to or even, according to some studies, lower than non-inhalers ". He adds (incorrectly): " Professor Burch asserts that this alone refutes the (causal) hypothesis . . .". This is pure invention. At the end of the section " Inhaling " I write on p. 356: " We are forced to conclude that the evidence for the effects of inhaling cannot provide definitive tests of all causal hypotheses, although it might help to define some of those anatomical sites where cigarette smoking does *not* exert a direct carcinogenic action ". (Original italics.)

As the author of a book that criticizes some of the key assumptions underlying much contemporary cancer research I had hoped for reasoned appraisal (and have not been wholly disappointed) although I was prepared for expressions of resentment. Is it unreasonable to expect that a reviewer should be familiar with, and make some reference to, the biology of cancer?

P. R. J. Burch
The General Infirmary, Leeds.

REFERENCES

Doll, R. & Hill, A. B. (1964) Mortality in Relation to Smoking: Ten Years' Observation of British Doctors. *Br. med. J.*, i, 1399 and 1460.

Hammond, E. C. (1966) Smoking in Relation to Death Rates of One Million Men and Women. *Natn. Cancer Inst. Monogr.*, **19**, 127.

Peto, R. (1976) Handout at Specialist Course, Royal Postgraduate Medical School, London.

Todd, G. F. (1972) *Statistics of Smoking in the United Kingdom.* 6th Ed. London: Tobacco Research Council.


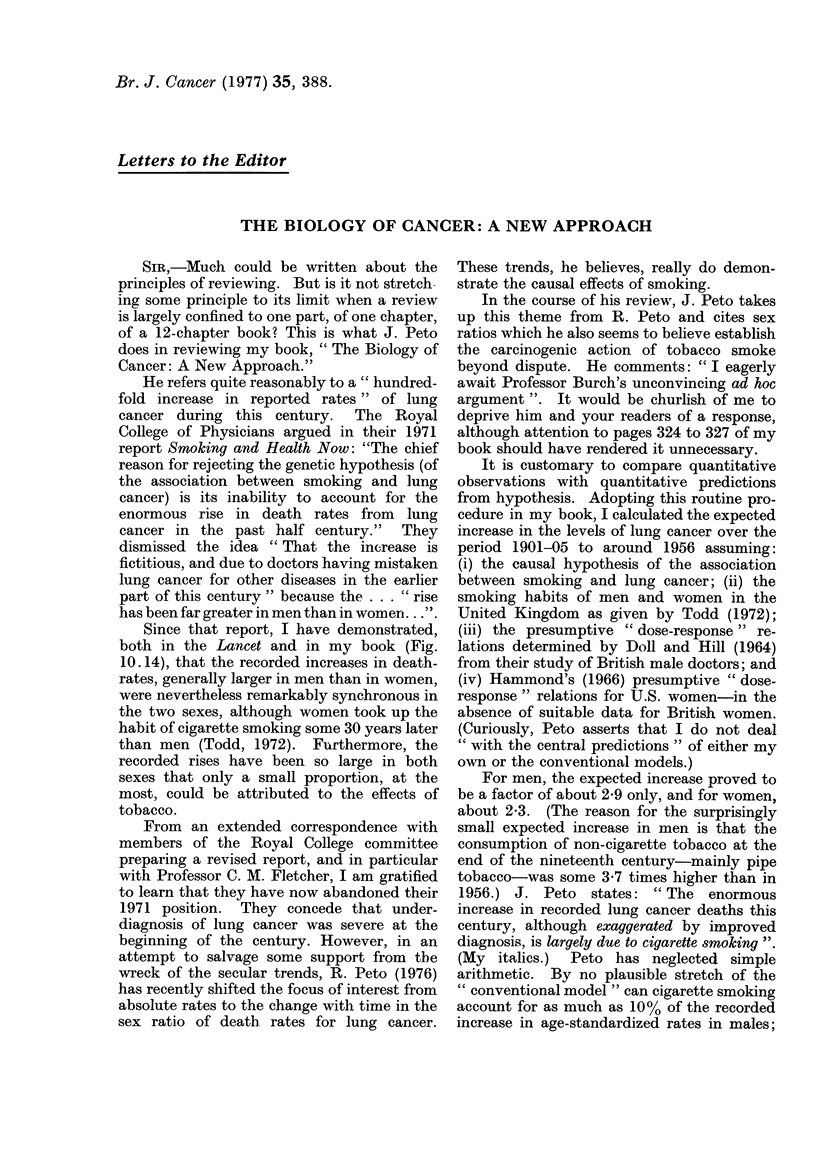

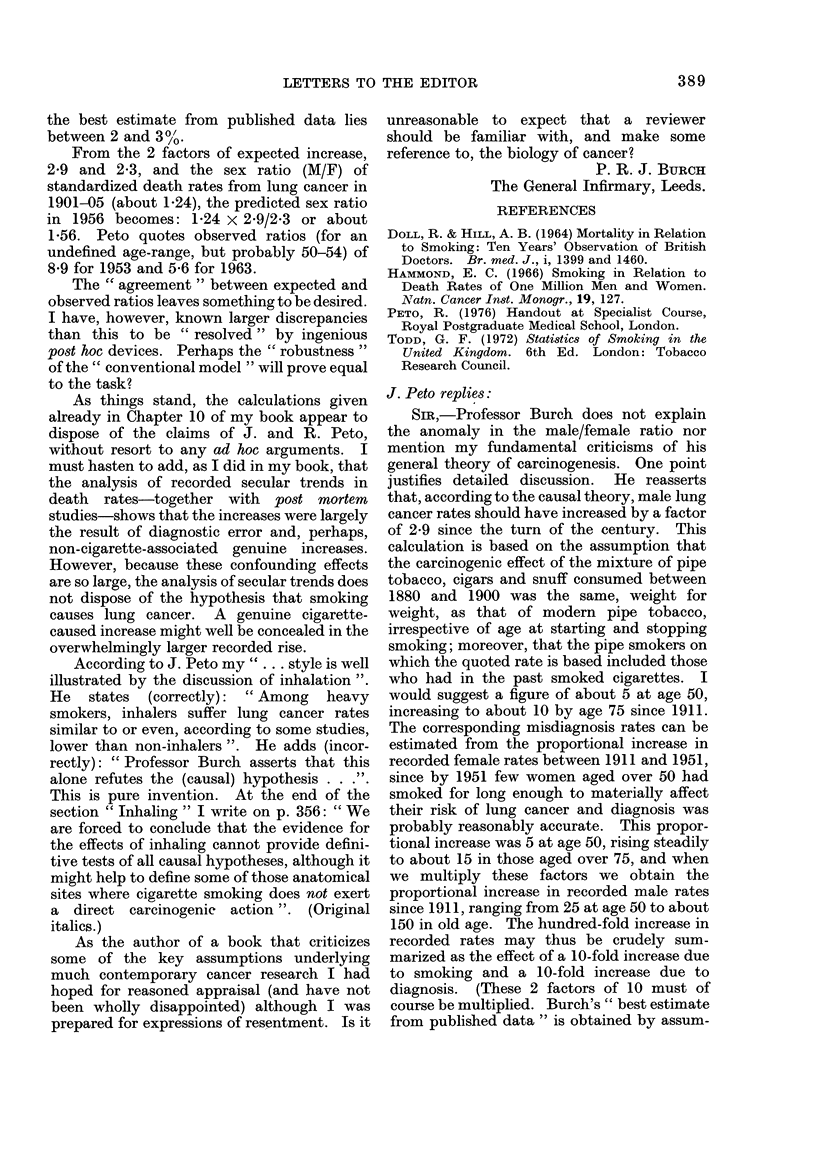


## References

[OCR_00184] DOLL R., HILL A. B. (1964). MORTALITY IN RELATION TO SMOKING: TEN YEARS' OBSERVATIONS OF BRITISH DOCTORS.. Br Med J.

[OCR_00189] Hammond E. C. (1966). Smoking in relation to the death rates of one million men and women.. Natl Cancer Inst Monogr.

